# A Frequency Pattern Mining Model Based on Deep Neural Network for Real-Time Classification of Heart Conditions

**DOI:** 10.3390/healthcare8030234

**Published:** 2020-07-26

**Authors:** Hyun Yoo, Soyoung Han, Kyungyong Chung

**Affiliations:** 1 Department of Computer Engineering, Gachon University, Seongnam 13120, Korea; rhpa0916@gmail.com; 2 Department of Nursing, Yonsei University Wonju College of Medicine, Wonju 26426, Korea; hsy79@yonsei.ac.kr; 3 Division of Computer Science and Engineering, Kyonggi University, Suwon 16227, Korea

**Keywords:** deep neural network, big data, artificial intelligence, data mining, heart rate, healthcare

## Abstract

Recently, a massive amount of big data of bioinformation is collected by sensor-based IoT devices. The collected data are also classified into different types of health big data in various techniques. A personalized analysis technique is a basis for judging the risk factors of personal cardiovascular disorders in real-time. The objective of this paper is to provide the model for the personalized heart condition classification in combination with the fast and effective preprocessing technique and deep neural network in order to process the real-time accumulated biosensor input data. The model can be useful to learn input data and develop an approximation function, and it can help users recognize risk situations. For the analysis of the pulse frequency, a fast Fourier transform is applied in preprocessing work. With the use of the frequency-by-frequency ratio data of the extracted power spectrum, data reduction is performed. To analyze the meanings of preprocessed data, a neural network algorithm is applied. In particular, a deep neural network is used to analyze and evaluate linear data. A deep neural network can make multiple layers and can establish an operation model of nodes with the use of gradient descent. The completed model was trained by classifying the ECG signals collected in advance into normal, control, and noise groups. Thereafter, the ECG signal input in real time through the trained deep neural network system was classified into normal, control, and noise. To evaluate the performance of the proposed model, this study utilized a ratio of data operation cost reduction and F-measure. As a result, with the use of fast Fourier transform and cumulative frequency percentage, the size of ECG reduced to 1:32. According to the analysis on the F-measure of the deep neural network, the model had 83.83% accuracy. Given the results, the modified deep neural network technique can reduce the size of big data in terms of computing work, and it is an effective system to reduce operation time.

## 1. Introduction

With the development of the IoT industry, wearable equipment for collecting bioinformation has been universalized. The wearable equipment has overcome technical limits through performance improvement, lightweight, and increased battery lifespan, and its infrastructure has been expanded in combination with smartphones, tablet PCs, and other communications devices. In addition, with the advent of intelligent healthcare systems such as the smart home system, it is easy to access personal biodata. Aside from that, with the advancement of Health information system (HIS) in large hospitals, massive bio and health data are created and saved. Accordingly, the size of the generated health data is remarkably on the rise, reaching the scale of big data. However, the current healthcare system utilizes health big data mainly for treating diseases of hospitalized patients and acute diseases, so that it has a lack of services in terms of the roles and preventive activities of chronic disease patients, integrated treatment and care, and personalized service [1]. A healthcare system needs to be expanded to the smart health service using life big data mining in order for chronic disease patients to care for themselves [2]. Therefore, it tries to develop to provide the health service for preventing recurrences of chronic diseases, such as diabetes, hypertension, dyslipidemia, cardiovascular disorders, and cerebrovascular disorders, and achieving early detection and care of stress and depression symptoms [3,4]. In addition, through the context recognition system, it is necessary to integrate the IoT based bio log big data individually obtained with a variety of health records and to analyze the data in an intelligent technique and provide customized information [4,5,6,7]. Recently, active research has been conducted on the current neural network-based health model in which recurrent connections of sequences are effectively learned to recognize sequential context knowledge and extract causal relations. That makes it possible to advance the healthcare service of the aging-affinity industry by monitoring a high-risk group of diseases as well as the long-term complications associated with chronic disease treatment, adverse effects, potential risk management of latent diseases, and by applying the health platform for the quantitative evaluation of latent severity to welfare facilities for senior citizens for managing and improving their chronic diseases.

This study proposes the deep neural network-based frequency pattern mining model for classifying heart conditions in real-time. As a personalized cardiovascular disorder prevention model, the proposed method cleans and processes the biodata collected from IoT-based biosensor devices, and it effectively classifies the input data into normal people, patients, and noise conditions. It consists of the fast Fourier transform-based power spectrum frequency preprocessing and the efficient combination with a deep neural network system. In the proposed method, a combined hybrid algorithm can improve an integrated learning model and can provide an optimized cardiovascular disorder risk prevention model with a high prediction rate.

This study is organized as follows. Section 2 describes the related researches of the relations between ECG patterns and cardiovascular disorders and machine learning and artificial neural network models. Section 3 describes the proposed a frequency pattern mining model based on deep neural network for real-time classification of heart conditions. Section 4 describes the experiments and result. Section 5 provides a conclusion.

## 2. Related Research

### 2.1. Relations between ECG Patterns and Cardiovascular Disorders

The heart regularly and repeatedly has systolic and diastolic movements for blood circulation. Cardiac pumping occurs with the systolic pressure of cardiac muscles. An Electrocardiogram (ECG) records the induced and amplified potential distribution difference of the electrical current occurring in the systolic pressure of cardiac muscles in the human skin. An ECG has the repetition of a certain form, and the cycle of the repetition for a certain time is named a frequency. The increase in the frequency is very closely related to exercise, pregnancy, or a stressful situation. The decrease in the frequency is related to a variety of critical pathological conditions, such as hypertension, hemorrhagic shock, and septic shock [8].

In an ECG graph, the P wave occurs in atrial depolarization, the QRS wave in ventricular depolarization, and the T wave in ventricular repolarization. The electrical activity of the heart begins from the sinoatrial node, spreading electrical stimulation to atriums and making atrial depolarization and the P wave. After that, the electrical stimulation reaches the atrioventricular node and is transmitted to the ventricles of the heart. When the ventricles of the heart are systolic, the QRS wave occurs. The T wave represents the recovery phase after ventricular systole. In the normal systolic and diastolic pressure of the heart, the R-R interval is classified as one cycle of heartbeats [9]. In particular, with the use of the R-R interval of ECG waveform, it is possible to diagnose arrhythmia and coronary arterial heart disease effectively. An arrhythmia occurs when the tissues generating the electrical stimulation of the heart or the stimulus conduction tissues transmitting a stimulus to cardiac muscle cells have a problem and therefore the regular systolic and diastolic pressure of the heart fails [10]. On balance, if a cardiac impulse is abnormally slow or fast, or irregular, it is diagnosed as arrhythmia. In terms of arrhythmia, the heart rate representing R-R interval should be lower than 60 at least. If it is lower, it is called bradycardia. If the heart rate is 100 or more, it is called pyknocardia [11].

Based on ECG, it is possible to analyze abnormal activities of the heart, such as Bundle Branch Block (BBB), Premature Ventricular Contraction (PVC), and Premature Atrial Contraction (PAC) [12,13,14]. Bundle branch block means the block of the electrical transmission in the right bundle branch or left bundle branch of the heart. It is found when one innately has an atrial septal defect, hypertension, ischemia heart disease, or cardiomyopathy in an early stage. The PVC is a common type of arrhythmia. If a patient who has a history of relevant illnesses has PVC, it is possible to cause ventricular tachycardia. PAC can trigger such complications such as cerebral infarction or systemic emboliform. In terms of arrhythmia, BBB, PVC, and PAC are very important factors to detect and predict cardiovascular disorders [13,14]. Table 1 presents the duration and related diseases by type of ECG waveform.

In the previous study on the ECG based frequency analysis, there is the clinical result that ECG can reflect the change of the automatic nervous system beyond the physiology of the heart [15]. In the frequency spectrum analysis related to stress, the frequency bandwidth related to stress is in the low frequency (LF) area, or 0.04–0.15 Hz. The frequency in the bandwidth is related to the sympathetic neural activity of the automatic nervous system, and it increases under stress [16]. When stressful situations occur, the brain releases various hormones, including adrenaline and cortisol, to recognize stress and respond to major stresses. During an acute stress situation, the heart beats faster and blood pressure rises under the influence of these hormones. In addition, fast breathing, increasing body temperature, and lots of sweating occur. Emotionally, anxiety and sensitive feelings arise, and additional symptoms such as headaches occur [17]. The most basic way to measure stress-related hormones is blood tests. However, the collection of blood requires a particular place or equipment and causes adverse effects such as pain and neurological damage. Most of all, it is possible to obtain the test result long after a symptom occurs. In responding to stress, it is important to escape immediately from a stress exposure circumstance. Accordingly, it is very useful to measure the change in the heartbeats which is made by stress derivatively [18]. The rate and frequency of pulse are very different depending on age, sex, and exercise even in the normal rest state. Therefore, to measure the change in pulse frequency and to discriminate an increase or decrease in the frequency, it is necessary to learn resting heart rates in advance. Therefore, by learning clinical data in advance depending on various diseases and situations, it is necessary to establish a model for discriminating similar relations.

### 2.2. Machine Learning and Artificial Neural Network Models

Machine learning is a process of analyzing associations after a series of pre-training data are learned in order to solve a problem. The basic structure of machine learning is divided into the step of making a model fitting application from the basic training data and into the step of repeatedly learning and updating data in the way of applying the data to a real environment. By using training data as the input of a virtual learning system, it is possible to create a learning model. Accordingly, it is possible to draw an outcome intelligently without designing a separate algorithm in line with each situation [19,20]. Machine learning is classified into supervised learning and unsupervised learning according to whether there are learning data. Supervised learning constructs associations with the uses of pre-training data and its target result as input and extracts an output when new data come in through the associations. Unsupervised learning finds the relations between input data. A simple type of supervised learning utilizes a multivariable linear regression algorithm to establish a single-layer neural network and thereby calculates a weight in the equation of the straight line and predicts an outcome [21]. In reality, the associations of data can be very complex, and fail to always converge to a continuous and one-dimensional straight line. Therefore, such supervised learning uses multi-layer neural network algorithm. The structure of a multi-layer neural network consists of three parts. The first one is the input layer that receives input data. The second one is multiple hidden layers in which association weights are saved. The last one is the output layer that displays output data. In a multi-layer neural network, the depth of hidden layers become deep depending on problem complexity [22]. A general multi-layer neural network connects individual nodes through their inputs and outputs so as to make a structure with multiple layers. In particular, the multi-layer network with multiple hidden layers is called a deep neural network.

To extract the learning outcome of the neural network, it is necessary to add and process the data received from multiple input nodes to an output node. In the whole model, weight is adjusted depending on input strength in order for repeated learning. Weight is a tool for the memory of a node and represents the importance of each node. If a new input value comes in after learning is complete, a proper output value is extracted according to connection strength. The process of updating a weight from the input layer to the output layer and bringing a result from an activation function is named forward propagation. In the forward propagation process, the value of the result generated in the output layer has a difference (error) from an actual value. Backpropagation is one of the methods of sending the difference in a reverse direction in order to calculate the weight. The cycle of learning all learning data is called one epoch. With a rise in the epoch, weight continues to be learned and the error gradually reduces. In a deep neural network, numerous nodes are connected with each other so that it is necessary to calculate the weights of the nodes. For this reason, an efficient method of calculating weight is required. As a universal weight calculation technique, there is gradient descent. In particular, deep neural network among artificial neural networks has a structure with multiple hidden layers. A deep neural network model processes large structured or unstructured data that exceed a conventional database processing capacity and extracts meanings. Therefore, in the big data-based machine learning, a deep neural network model combines multiple layers of the neural network and draws a meaningful outcome through efficient reasoning and learning. The learning structure of the deep neural network is suitable for massive learning data, and learning is made from the observed items, and thereby a proper approximate function can be created [23,24]. A deep neural network is favorable to the modeling of complicated nonlinear relations and is much used in the learning and recognition area. In addition, to solve a problem, it is applicable to diverse areas, such as image processing, object recognition, tracking, and text and voice recognition, and its performance is recognized to be excellent. A deep neural network can have a structure of object identification model or voice recognition, in which each object is expressed with the hierarchical structure of its basic factors. Additional layers can collect the features of their lower layers. Thanks to the advantage, a deep neural network, compared to neural networks with similar structure, can model various structure types of data only with small units. The artificial neural network with the structure requires a lot of time for learning. Additionally, it has difficulty with actual use due to its partial optimization, as well as the problem of over-fitting to pre-training data. In machine learning with high complexity, the more higher hidden layers there are, the more operation cost increases exponentially. With the remarkable rise in the size of big data, the operation cost for the data also increases. That is an obstacle to actual use. As a solution to the problem, GPU is used as the H/W of the computing system, or data-parallel processing or model parallel processing is developed as S/W [25,26]. With the improvement in the high-performance parallel computing capacity based on GPGPU (General Purpose of Graphic Processor Unit), the operation cost is also improved greatly. As various processing techniques are improved, big data are secured, and hardware is outstandingly developed, the problems with practical use are solved. As a result, a deep neural network is applied to a variety of convergence areas. The deep learning-based artificial intelligence system improves its performance positively in such areas as image processing, voice recognition, and natural language processing. Recently, products reflecting deep learning technology, including voice recognition speaker and interpreter, are released. As such, technology is fast developed. With the use of the deep learning-based data analysis, spam e-mail filter, stock price prediction, Facebook advertising, recommendations of Amazon, Google Trend, profiling, data sports, presidential election prediction, etc. are developed. In addition, the IoT and big data processing technology helps to provide diversified and personalized healthcare services by finding user health information and dietary habit patterns [27,28].

## 3. A Frequency Pattern Mining Model Based on Deep Neural Network for Real-Time Classification of Heart Conditions

### 3.1. Preprocessing Using Fast Fourier Transform

To evaluate the current risk factors reflected in real-time, it is necessary to establish a bioinformation processing system using IoT healthcare systems. The biosignal data collected through bioinformation are divided into numerical biosignal and time-series biosignal data. Numerical biosignal data include height, weight, body temperature, and level of blood glucose and are discrete numbers obtained in a particular point [29,30].

There are two basic methods of obtaining biosignals from the heart. First, there is an Ultrasonography using ultrasound and reflected waves, and the second is an Ectrocardiography (ECG) method that measures the electrical activity of electrodes by attaching them to the skin of the body. Relevant mobile devices utilize a variety of methods, such as the infrared and optic sensor-based method of measuring a blood flood rate on a fingertip, an easy-to-measure body region, and the method of using the sensors attached to clothing. Therefore, the type of biometric information acquired depends on whether the personal measuring device is used. Even if the measuring equipment with the same functions is utilized, various problems such as low resolution, missing, and noise can occur. For this reason, it is necessary to apply a variety of resolutions and the preprocessing technique robust for missing data. The study utilized ECG sensors that were easy to access and could be used as wearables [31,32] and used a pulse sensor input device for the basic data that were needed for qualitative and quantitative analyses and comparison of data processing models.

The data measured from a pulse sensor can be designed in a form of frequency. There are various techniques for determining whether heartbeats are normal with the use of characteristics of frequency. The Fourier transform for frequency analysis transforms a time-domain function to the frequency factors and is a representative transform method used in various areas including signal processing, voice, image, and communications. The frequency bandwidth collected in this way can be analyzed, and feature extraction and noise filtering are made possible. In particular, the Fourier transform is applied to data reduction. If a deep neural network system directly analyzes the input data of a sensor, the input layer can be more complicated in proportion to input data construction, and the operation amount of the deep neural network can increase exponentially. Therefore, the input data reduction technique is essential for implementing the terminal system of an actual model.

Generally, the Fourier transform is unable to analyze the association of frequency with the change of time. The data received from a sensor are discrete values; thus, it is necessary to apply a discrete Fourier transform. For this reason, this paper applies the fast Fourier transform in which a frequency extraction window size is fixed and the influence with the change of time is effectively expressed. As an efficient implementation method of discrete Fourier transform, a fast Fourier transform removes repeated operations in transform so as to improve its transform speed [33]. In the condition that a discrete signal input function is G(x), the equation of fast Fourier transform is presented as Equation (1):

(1) $$Ft\left(u\right)=\overset{N-1}{\underset{n=0}{\sum }}G\left(x\right)e^{-i\frac{2\pi ux}{N}}$$

In the equation, *N* represents the total number of data and *i* represents an imaginary number [33]. If $e^{-i\frac{2\pi ux}{N}}$ is defined as *W_N_* and *N* defined as 2*K* in the equation, the equation can be written as in Equation (2) in the separation of even and odd terms.

(2) $$Ft\left(u\right)=Ft\_even\left(u\right)+Ft\_odd\left(u\right)W_{2K}^{u}$$

Even-term is defined as Ft_even(u), odd-term is defined as  Ft_odd(u), and the cycle of each term is *N/2*. Therefore, if *u + K* replaces *u*, the value has no change and only the sign of *W_N_* is changed. Accordingly, if *u + K* substitutes for *u* in Equation (2), the equation can be written as Equation (3):

(3) $$Ft\left(u+K\right)=Ft\_even\left(u\right)-Ft\_odd\left(u\right)W_{2K}^{u}$$

If *N = 2K* number of data is changed with the use of Equation (3), it is possible to calculate the total extraction value with the uses of *K* number of even extraction values and odd extraction values. Since recursive extraction is made possible in logic implementation, the method is more effective [33]. Therefore, it is possible to analyze associations of time and frequency in real-time on the basis of the rapidly changing frequency data.

To use the fast Fourier transform, it is necessary to normalize the pulse frequency data received in real-time [18]. If the fast Fourier transform is generally applied for frequency analysis, it is necessary to set the endpoint of the transform start point. Typically, the Pan–Tompkins algorithm based on ECG gradients [34] is used to detect R-peak. If a neural network system is used, it is possible to learn various types of power spectra and therefore add a hidden layer of the neural network. The size of the sampling data should be larger than the maximum size of abnormal R-R waves. Based on bradycardia (a heart rate of fewer than 60 beats per minute), an analysis time should be at least one second. In consideration of a user’s response time, a delay time should not be over several seconds. An ECG sensor generates 100 data per second, and a fast Fourier transform performs fast operation with the data size 2n. This study sets the base time to 2.56 s. In this case, 356 times of discrete data sampling cycles are used, and, even if the window of sampling data goes out of P-U waveform, a part of its front and back waveform is always included. Therefore, in a deep neural network, it is possible to learn the characteristics of each waveform. The first generated result of the fast Fourier transform is bi-symmetrical data. Accordingly, the left-side data are obtained and converted into the power spectrum after the addition of square operation. For noise filtering, error ECG data generated by sensor initialization and malfunction are removed, and noise is removed by the band-pass filter of the power spectrum. To improve the ECG frequency analysis method used in previous research, this paper supplements the three-section separation method based on low- and high-frequency separation [16] and classifies frequency data into eight sections. The extracted result can have a difference in the overall input frequency depending on the characteristics of the sensor and a wearable position. Therefore, a relative frequency ratio is used. As a result, the cumulative frequency percentage of frequency sections of the power spectrum is used as the input data of deep neural networks.

### 3.2. Real-Time Frequency Pattern Acquisition and Power Spectrum Extraction

To implement ECG waveform as the ECG data collected for analyzing heartbeats, it is necessary to use the pulse data collection system using ECG biosensor. As a pulse sensor for measuring heartbeats, an AD8232 chip was used. The AD8232 chip is a portable ECG measuring device developed for monitoring healthcare fitness and activity pulses and is used to obtain biopotential signals. It can extract, amplify, and filter signals in noise circumstances and runs with the 170 μA ultra-low power. With the use of an external battery, it can become small or modularized. Its common-mode rejection ratio is 80 dB, and the amplifier factor is 100 times. The internally converted digital value is transmitted at the speed of 9600 bps. As an Arduino system for processing the collected basic data, Arduino Uno (R3) was utilized. Arduino Uno is a microcontroller board with ATmega328 based MCU, featuring 16 MHz speed and 32 Kb memory. For data transmission and reception, the HC-05 Bluetooth module was applied. The Bluetooth module has an IEEE 802.15.1 protocol and its performance fits sensor data transmission and reception. Compared to XBee [18,31], Bluetooth supports data transmission and reception without any installation of a separate module in the reception part and makes it possible to implement a low-power system.

**Figure 1:**
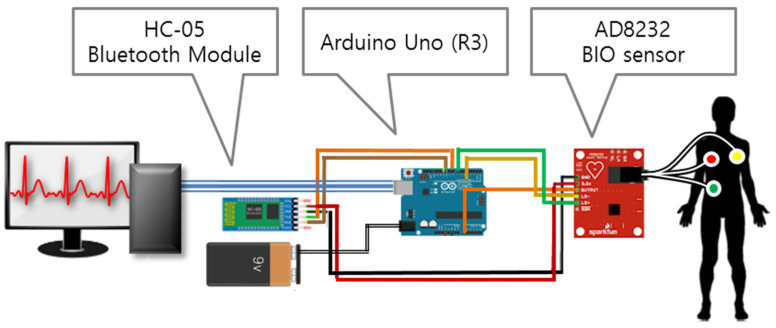
Composed pulse measuring equipment.

As a development tool, ARDUINO 1.8.7(ARDUINO, Turin, Italy) was used. The device made is similar to a wearable clothing system. The bioinformation measuring device implemented is shown in Figure 1.

**Figure 2:**
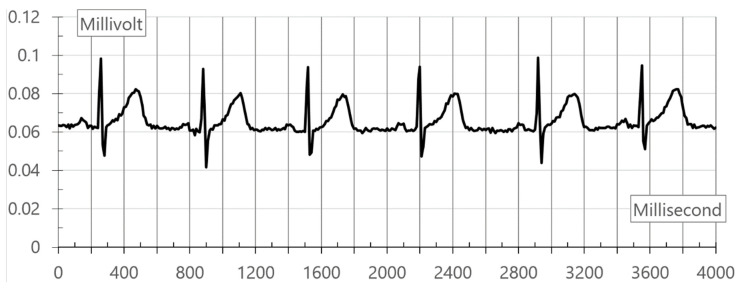
Collected ECG packets graphs.

An AD8232 chip can cause the delay time of several seconds due to its initialization and the optimization of its internal algorithm. It transmits ECG data at the speed of 100 Hz. If the collected data are out of the normal range (200–500), the error value is removed. Instead, the value of Millivolt is saved in the database. The collected ECG packets graphs are shown in Figure 2.

**Figure 3:**
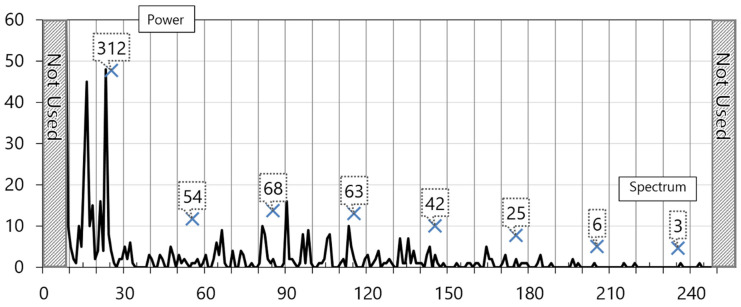
Graph of power spectrum extracted from normal ECG data by fast Fourier transform.

The collected ECG packets are transmitted to a terminal server. A fast Fourier transform is executed in the terminal. The left-side part of the transformed power spectrum is used. The width of the generated power spectrum is extracted with 256 discrete values. For the removal of noise, 240 sections except for the lower eight sections with rapidly high frequency and the upper eight sections with almost no frequency are used. The 240 sections are divided into eight parts, and the cumulative value in the unit of 30 is used. Therefore, it is finally organized into eight data. This method supplements the three-section separation technique of simply classifying frequency data into low and high frequency [16] and generates more frequency classification information. Accordingly, the generated information can be used as the neural network learning data that have various meanings. Figure 3 shows the graph of the power spectrum extracted from normal ECG data by the fast Fourier transform.

**Figure 4:**
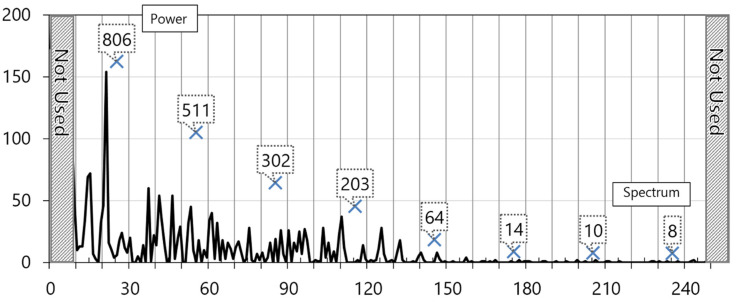
Graph of fast Fourier transforms power spectrum from abnormal ECG.

The left and right diagonal lines of the graph are removed. The X mark in the unit of 30 means the cumulative value of spectrum strength after the segmentation of sections except for noise sections. The accumulated value is eight, which is used as input data of the deep neural network. Figure 4 shows the graph of the fast Fourier transform power spectrum of the abnormal ECG from an 82-year old-patient with heart valve disease.

To use data as the input data of deep neural networks, it is necessary to convert them into a relative ratio. In the condition that the cumulative value of total spectrums is *AF* and the cumulative value of each section is *PF*, the ratio *R* is calculated in the equation *R = PF/AF*. With the equation *R*, the input data of each node in the input layer of the deep neural network do not exceed “1”.

The generated bioinformation data become the basic data for learning the semantic connection between heartbeat conditions and human activities and objective context recognition modeling in a neural network model. The total size of the data to transmit per device in the model is ~15 KB. For pulse data, 256 data are collected and transmitted at an interval of 2.6 s. A terminal module transmits 10 virtual pulse data. In a deep neural network, the data processing capacity per hour is 360,000 per person. In a simulation situation, 3,600,000 data per hour should be processed. To use a deep neural network model, it is necessary to apply the first learning process. For a variety of classifications, it is required to provide a diversity of data based on users’ stable pulse frequency, sleep, exercise, resting, and diseases. For easy performance evaluation, biodata are classified into normal, abnormal, and noise data types on the basis of the basic data in the resting state and are learned. A proper learning volume and rate are adjusted according to the experimental result.

### 3.3. Deep Neural Network Configuration for Classification of Heart Conditions

To evaluate the state of heartbeats, it is necessary to use a technique of learning and identifying each situation. Since many biodata are generated in real-time, machine learning is performed in the bioinformation processing technique for big data processing. The deep neural network algorithm of machine learning is useful for data classification and clustering and has strong identification of data change. A neural network algorithm is capable of analyzing the risk of ECG data and discriminating between exercise or resting pulse situations and emergency situations [35]. A deep neural network algorithm needs to learn the basic situations and disease data prior to the operation procedure of the user, and to make an approximate function with high accuracy on the basis of the learning. The main factors for implementing a deep neural network model are training and inference. Training begins with the process of entering data in the first input layer of the neural network. After that, the feed forwarding process is executed for calculating the weight of each node. This operation is made up of the process of calculating weight from a hidden layer to an output layer [36]. After that, the weight is modified according to the accuracy of the result in the error correction step. The learning process of the deep neural network is divided into supervised learning and autonomous learning and is selected differently depending on a given issue.

**Figure 5:**
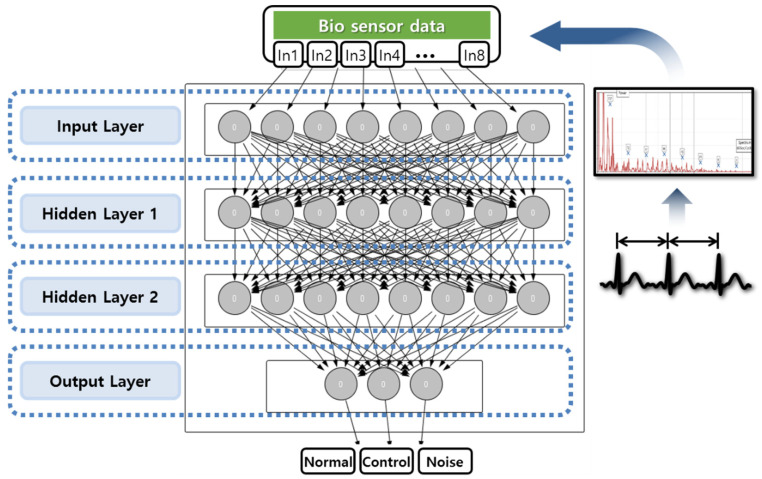
Internal structure of deep neural network model.

For the analysis of the risk of pulse frequency, supervised learning is applied. Given a large load of neural network learning in actual use, it is necessary to build a modularized and concise model, rather than a universal model. Therefore, the deep neural network to implement has the structure of the input layer, output layer, and hidden layer in the minimum units. The internal structure of the deep neural network model is shown in Figure 5. The preprocessed bioinformation data are divided into eight sections, and the appearance count of each frequency bandwidth is set as an input layer. Accordingly, the input layer of the neural network has eight neurons [21], and three-stage hidden layers have twenty-seven hidden nodes. A hidden layer learns the power spectrum that changes differently depending on a variety of window positions of sampled frequencies. The output layer has three neurons to compare and evaluate three types of results: normal, abnormal, and noise.

For the data learning of neural networks, stable pulse, normal pulse, and abnormal pulse data and data in noise state are used. The data obtained in a generally stable state are applied, and preprocessed frequencies are sampled and learned. A deep neural network system needs the weight of minimizing a difference between the output value and the actual value of each node. For an accurate weight, it is necessary to apply a technique of finding the minimum error of neurons. Basically, a weight is calculated and updated by gradient descent. Gradient descent calculates an error of input value with the use of a gradient and moves in the direction of the steepest descent of gradient. It makes repeated calculation until an error reaches the minimum value and updates connection strength in the direction of minimum error. To find a minimum error, the cost function is needed. If the target output for the output value b is a, the cost function $C_{p}$ for nth power spectrum data is written as Equation (4):

(4) $$C_{p}=\frac{1}{2}\underset{t}{\sum }{\left|a_{nt}-b_{nt}\right|}^{2}$$

$a_{nt}$ means the target value of the tth factor for nth power spectrum data. $b_{nt}$ represents the output value of the tth factor for nth power spectrum data. To find the function f(x) to optimize an error point, it is necessary to use Equation (5) that presents the relation between the target values $x_{i+1}$ and $x_{i}$.

(5) $$x_{i+1}=x_{i}-\alpha \nabla f\left(x_{i}\right)$$

∇ means a gradient and α is a learning rate. As the value of α becomes larger, it is possible to approach a result value more quickly [33]. However, error vibration occurs. The method re-operates all data and nodes so that much computing power is consumed. Nevertheless, it is favorable to a simple analysis model due to the relatively simple implementation of the method. As an improved technique, there is backpropagation that corrects an error of nodes in the reverse direction with the use of a differential function [37]. The method finds a loss function on the basis of the final output data and adjusts the weight of each node in order to make a proper value displayed in an input layer. It goes in the other way of the forward propagation and runs back from the output to input through chain rule.

As for the development of the deep neural network model for health big data processing, it is necessary to do modeling in the way of changing the size of layers, the complexity of error reduction operation, and a learning rate of each node. Through repeated testing, it is possible to make the most appropriate recommendation model. A data amount and the count of learning remarkably increase in proportion to the number of nodes used according to the size of the deep neural network model made, and thereby the learning time lengthens exponentially. In particular, the more hidden layers there are, the more the cost for connection weights increases exponentially. Therefore, based on the hidden layer with high importance and the count of learning, it is necessary to make a structure with the optimal performance for operation cost.

The deep neural network system goes through the preprocessing-based data classification, the transmission of the classified data to a terminal, and training of the transmitted data through operation processing. The completed system transmits the data requiring discrimination to a neural network terminal. The neural network system transmits the extracted result to a server. In this way, it is possible to infer and determine the new input data of each node.

### 3.4. Configuration and Processing of Heart Condition Classification System

**Figure 6:**
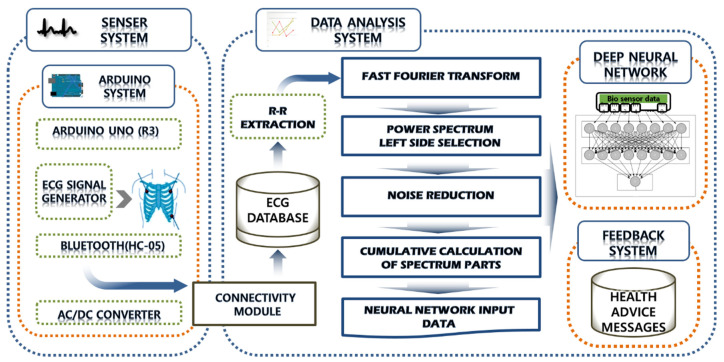
Structure of terminal models including the bioinformation collection module.

In order to consistently store, identify, and manage the collected data, need to apply an integrated data schema. Biometric information is collected in real-time and processed in a terminal, and the processing results are stored in separate table using fast Fourier transform regardless of the data received and client request. The structure of the terminal models including the bio information collection module is shown in Figure 6.

The basic process of the learning model is implemented by adjusting the weight and learning repeatedly through the collection. The deep neural network model used in this study specifies the set result from training data and utilizes the supervised learning technique using feedback evaluation. Eight input nodes are configured according to the frequency input. A deep neural network system consisting of multiple hidden layers with the same type of hidden nodes is established. The established neural network makes it possible to model a complicated nonlinear relation.

To provide information service for users, the final output model makes communication through the web server. In this circumstance, users are able to have online services easily. The server system has the H/W specification of i5-6600 3.30 Ghz CPU (Intel^®^, Santa Clara, CA, USA) and 8 GB memory, and the terminal device for sensor data collection and operation has the H/W specification of Intel^®^ Celeron N2930 1.83 Ghz CPU and 4 GB memory. A compiler language for S/W implementation needs various libraries for serial communications, web service, and DB connection. The Java version for the development is Java(TM) SE Runtime Environment (build 1.8.0_111-b14) (Oracle, Redwood City, CA, USA). As a development tool, IntelliJ IDEA Build: 191.6707.61(JetBrains, Prague, Czech Republic) was used. As external libraries, MySQL-connecter-java-8.0.15(Oracle, Redwood City, CA, USA) for DB access and RXTXcomm (64bit) for serial communications were used. Apache Tomcat 9.0 was used as an HTTP web server for communications and web service. To establish a reliable neural network model, the code of Java Deep Learning Essentials was used as a reference [38,39]. In the code, each layer is set as a class. For a deep neural network, multiple hidden layer objects are generated. As a DBMS, MySQL Ver 8.0.15 was used. As a DBMS tool, MySQL Workbench Ver 8.0.15 was used. In terms of the implementation and evaluation, the total ECG database use volume for implementing fast Fourier transform and deep neural network is 41.46MB, and about 50,000 pulses basic data account for 82% of storage.

**Figure 7:**
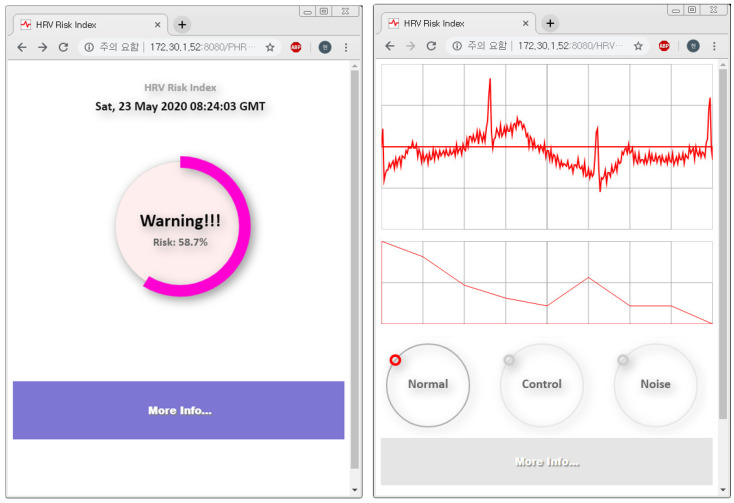
Result of the web service screen for providing risk index of cardiovascular disorder.

If the risk of the finally constructed cardiovascular disorder is high, the button for going to the ECG information screen is created. On the screen, it is possible to find the window of pulse information. On top of the pulse information window are pulse situations and a graph of the total frequencies after fast Fourier transform. On the bottom of the window is the result of the normal, abnormal, or noise evaluation in the deep neural network. Figure 7 shows the results of the web service screen in which the risk information about cardiovascular disorders and the collected pulse information are displayed.

Through the service, a user is able to judge the risk index of its cardiovascular disorder easily and conveniently according to the personal change in heartbeats and to obtain related information.

## 4. Performance Evaluation

If the input layer of the deep neural network system is complicated according to its input data construction, the complexity exponentially increases the operation volume of the system. In an actual environment, the response speed of the system should be immediate. For this reason, its computing power is very limited, and the use of big data platforms is generally restricted. Accordingly, an input data reduction technique is very important [40]. This study utilized the fast Fourier transform to reduce the data to be processed in real-time. From the first sensor, 256 data come in every 2.5 s, which are reduced to eight data after the constructed fast Fourier transform system and the cumulative count of each frequency is applied. Therefore, data reduction occurs at the ratio of 1:32.

For the initial settings of the neural networks, evaluation is made in the categories of accuracy and recall. Accuracy means a rate of correct answers for the total test results. Since accuracy is intuitive, it is most generally used. Accuracy is calculated as in Equation (6):

(6) $$Accuracy=\frac{TP+TN}{TP+FN+FP+TN}$$

TP, FN, FP, and TN represent the number of true positives, false negatives, false positives, and true negatives, respectively [41].

A deep neural network has different performance depending on a learning rate and a learning amount. Therefore, it is necessary to extract a set value that fits a system by changing the learning rate and learning amount. In particular, through gradient descent learning, a neural network finds the optimal weight that minimizes a prediction error. If a learning rate is too high, it is possible to continue to pass an optimal weight. If it is too low, it takes a lot of repeated learning and time. For this reason, it is important to set a proper learning rate. For neural network learning and evaluation, it is required to collect data from an ECG sensor [32] in a real environment. The collected data are divided into a normal group and into an abnormal group. The collected samples are from adults living in Wonju, Gangwondo. The ECG data records collected in three classes are presented in Table 2.

Data were collected in the condition that each person has their resting state for about 10 min. The data of the noise group were collected in the repeated recall of external vibration situations of a sensor. The total number of the initially collected ECG data was 560,000. Undersampling technique was used to solve the class imbalanced data problem. Therefore, it was based on the data size of the patient class that was difficult to acquire. It limited the size of the data of normal people, and the size of the noise group was also configured proportionally. The error data collected from a sensor were deleted, and then 490,000 data were selected. The data were applied to the fast Fourier transform. There were 1917 transformed data. Table 3 presents the use of the history of ECG data.

**Figure 8:**
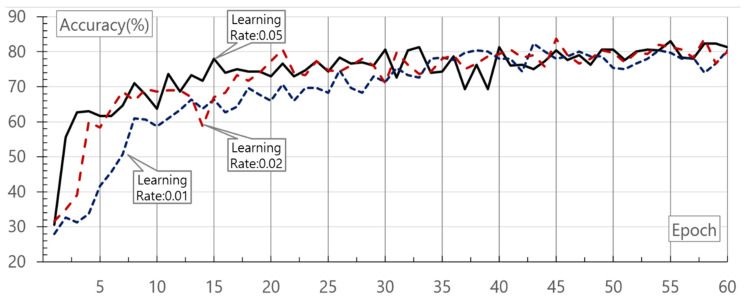
Result of accuracy evaluation according to learning rate and epoch of deep neural networks.

Finally, 1200 learning data and 300 data for evaluation tests were separately used. To avoid the problem of imbalanced data, data for evaluation were also written for each class on the same scale. In the circumstance, one epoch means 1200 times of learning. In addition, the input layer data of the neural networks were the normalized data that do not exceed “1” at most through preprocessing. Figure 8 shows the results of evaluating the learning rate and epoch accuracy in deep neural networks using the collected ECG data.

As shown in the evaluation result, accuracy increases along with a rise in the count of learning. Even though accuracy also rises along with an increase in the learning rate, there is no big difference in the learning of 40 epochs or more. The highest accuracy is 83.67% at the learning rate of 0.02 and in 45 epochs. Aside from that, it is important to evaluate the performance as to whether actual disease data input are missed according to an experimental purpose. Therefore, the evaluation for the recall was performed. The recall is a rate of true positives and calculated as in Equation (7):

(7) $$Recall=\frac{TP}{TP+FN}$$

**Figure 9:**
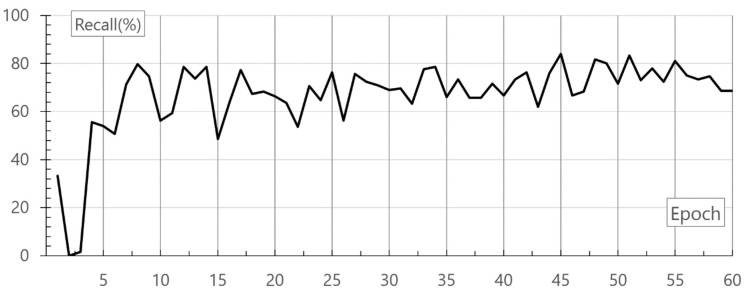
Result of recall evaluation.

TP and FN represent the number of true positives and false negatives, respectively. The evaluation for recall of the neural network was performed at the learning rate 0.02, separately. Figure 9 shows the result of recall evaluation and the randomly extracted learning and evaluation data. The result is the mean of the values measured repeatedly three times. As a result, in 45 epochs, the recall was 84.00%.

For the evaluation of deep neural networks, F-measure was compared. F-measure is a way of expressing precision and recall as one number. It is the harmonic mean of precision (P) and recall (R) and calculated as in Equation (8):

(8) $$F-measure=\frac{2\times P\times R}{P+R}$$

**Figure 10:**
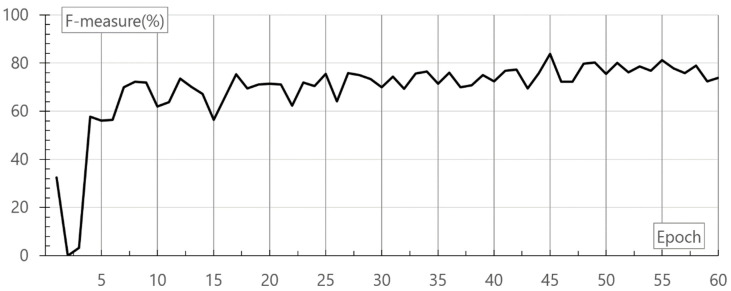
Result of F-measure according to the epoch of deep neural networks.

Figure 10 shows the F-measure result at the learning rate 0.02 according to the learning count of deep neural networks.

**Figure 11:**
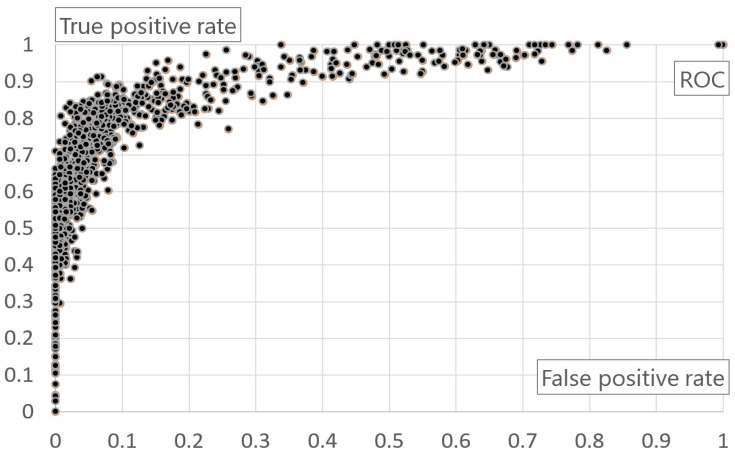
Result of ROC of deep neural networks.

As shown in the evaluation, in 45 epochs, the maximum F-measure is 83.83%. The completion time of 45 epochs is 171.9 s. A user system performs a service after learning is complete at the learning rate 0.02 and in 45 epochs. It takes 3.82 s per epoch. For the final evaluation of deep neural networks, ROC was compared. ROC is a performance evaluation technique for binary classifier systems. Figure 11 shows the final ROC result of deep neural networks.

Finally, the performance of the model was evaluated through a confusion matrix. Table 4 shows the result of the confusion matrix. The confusion matrix is the result of using 300 separately classified evaluation data. The reason the result of self-diagnosis of cardiovascular disorders is judged to be normal is that the disorders are controlled with healthcare and medication after the occurrence. The classification in the abnormal state for normal people seems to be influenced by local arrhythmia or pyknocardia.

In conclusion, a deep neural network makes it possible to analyze an abnormal pattern. It will be possible to identify a variety of cardiac disorders by obtaining clinical data.

## 5. Conclusions

With the economic improvement and IoT development, it has been required to develop a more advanced health data processing model to improve the scalability of healthcare. Different types of data processing models have their advantages and disadvantages. By trying to make convergence for overcoming the disadvantages, it is possible to improve accuracy or reduce operation resources. This study aimed at solving the conventional problems in the combination of fast Fourier transform and deep neural network models on the basis of pulse senor data and thereby improve data fitting and response speed. To evaluate the performance of the proposed model, this study evaluated a rate of data operation cost reduction. As a result, with the uses of fast Fourier transform and cumulative frequency percentage, the size of ECG reduced at the ratio 1:32. Therefore, the proposed method reduced the big data processing operation cost, secured accuracy, and improved a personalized healthcare service at a realistic level. For the telemedicine based on IoT equipment, the fast Fourier transform and neural network algorithm based learning were implemented and evaluated. The deep neural network model had 83.83% F-measure at the learning rate 0.02 and in 45 epochs. It showed the possibility that a healthcare model can be established with high accessibility and at a low cost. Given the result, the combination of various algorithms makes it possible to improve system performance. By offering a risk rate and risk pattern information, it is possible to provide a highly satisfying personalized service for a variety of users. Based on the information provided, user can conveniently check and judge the risk index of their cardiovascular disorders conveniently and thereby block or avoid the external circumstances that cause risk factors.

## Figures and Tables

**Table 1:** Duration and related diseases by type of ECG waveform.

Wave Form	Heart Activity State	Duration of Waveform (Second)	Relevant Disorders
P	Atrial depolarization	0.05–0.12	Acute myocardial infarction, sinus tachycardia, systemic hypertension, mitral or aortic valve disease, etc. Chronic obstructive pulmonary disease, asthma attack, acute pulmonary embolism, acute pulmonary edema, pulmonary edema associated with left ventricular dysfunction, etc.
QRS	Ventricular depolarization	0.06–0.12	Intraventricular conduction disturbance by bundle branch block, abnormal atrioventricular conduction (preexcitation), etc.
T	Ventricular repolarization	0.10–0.25	Myocardial ischemia, acute myocardial infarction, myocarditis, endocarditis, ventricular hypertrophy, administration of heart drug, etc. Electrolyte imbalance

**Table 2:** Record ECG data collected in three classes.

**Normal Group (Normal Person)**		
**Age**	**Sex**	**Disease**
38	F	-
43	M	-
65	F	-
66	M	-
74	F	-
**Control Group (Patient)**		
**Age**	**Sex**	**Disease**
48	M	Heart failure
80	F	Myocardial infarction
82	M	Valvular heart disease (Congenital)
83	M	Myocardial infarction
92	F	Valvular heart disease (Acquired)
**Noise Group (Noise State)**		
**Age**	**Sex**	**Disease**
38	F	-
43	M	-
48	M	Heart failure
66	M	-
80	F	Myocardial infarction
82	M	Valvular heart disease (Congenital)

**Table 3:** ECG data usage history.

	Data Kind	Collection Data	FFT Processing	For Learning	For Evaluation
Group					
Normal		197,222	770	400	100
Control		133,655	522	400	100
Noise		160,040	625	400	100

**Table 4:** Result of confusion matrix.

		**Prediction**		
		**Normal**	**Control**	**Noise**
Condition	Normal	68	25	0
	Control	19	79	7
	Noise	5	0	97

## References

[1] Penninga L., Lorentzen A.K., Davis C. (2019). A Telemedicine Case Series for Acute Medical Emergencies in Greenland: A Model for Austere Environments. Telemed. J. E-Health.

[2] Bala N., Price S.N., Horan C.M., Gerber M.W., Taveras E.M. (2019). Use of Telehealth to Enhance Care in a Family-Centered Childhood Obesity Intervention. Clin Pediatr..

[3] Golas S.B., Shibahara T., Agboola S., Otaki H., Sato J., Nakae T., Hisamitsu T., Kojima G., Felsted J., Kakarmath S. (2018). A Machine Learning Model to Predict the Risk of 30-day Readmissions in Patients with Heart Failure: A Retrospective Analysis of Electronic Medical Records Data. BMC Med. Inform. Decis. Mak..

[4] Kang J.S., Shin D.H., Baek J.W., Chung K. (2019). Activity Recommendation Model using Rank Correlation for Chronic Stress Management. Appl. Sci..

[5] Richesson R.L., Hammond W.E., Nahm M., Wixted D., Simon G.-E., Robinson J.-G., Bauck A.E., Cifelli D., Smerek M.M., Dickerson J. (2013). Electronic Health Records Based Phenotyping in Next-Generation Clinical Trials: A Perspective from the NIH Health Care Systems Collaboratory. J. Am. Med. Inform. Assoc..

[6] Marziniak M., Brichetto G., Feys P., Meyding-Lamade U., Vernon K., Meuth S.G. (2018). The Use of Digital and Remote Communication Technologies as a Tool for Multiple Sclerosis Management: Narrative Review. JMIR Rehabil. Assist. Technol..

[7] Chung K., Park R.C. (2020). P2P based Open Health Cloud for Medicines Management. Peer Peer Netw. Appl..

[8] Lee B.A., Jung S.H., Han G.S., Lee S.W., Hong Y.S. (2004). Significance of Shock Index in Hemmorrhagic Shock and Septic Shock Patients. Korean J. Malacol..

[9] Ye C., Kumar B.V., Coimbra M.T. (2012). Heartbeat Classification using Morphological and Dynamic Features of ECG Signals. IEEE Trans. Biomed. Eng..

[10] Behar J., Oster J., Li Q., Clifford G.D. (2013). ECG Signal Quality during Arrhythmia and its Application to False Alarm Reduction. IEEE Trans. Biomed. Eng..

[11] American Heart Association Inc. http://www.heart.org/.

[12] Chan A.D., Hamdy M.M., Badre A., Badee V. (2008). Wavelet Distance Measure for Person Identification using Electrocardiograms. IEEE Trans. Instrum. Meas..

[13] Chauhan S., Arora A.S., Kaul A. (2010). A Survey of Emerging Biometric Modalites. Procedia Comput. Sci..

[14] Wubbeler G., Stavridis M., Kreiseler D., Bousseljot R.D., Elster C. (2007). Verification of Humans using the Electrocardiogram. Pattern Recognit. Lett..

[15] Oh S.H., Whang M.C., Im J.J. (1997). A Study for the Discrimination of Visual Emotions using Heart Rate Variability. J. Ergon. Soc. Korea.

[16] Stein P.K. (2002). Assessing Heart Rate Variability from Real-World Holter Reports. Card. Electrophysiol. Rev..

[17] Lee H.Y., Ham B.J. (2013). Stress and Mental Illness. J. Korea Contents Assoc..

[18] Chung K. (2011). Vital Signal Monitoring Simulation System by Various Visual Stimulus. J. Korea Contents Assoc..

[19] Nasrollahi A., Deng W., Ma Z., Rizzo P. (2018). Multimodal Structural Health Monitoring based on Active and Passive Sensing. Struct. Health Monit..

[20] Dastjerdi A.V., Buyya R. (2016). Fog Computing: Helping the Internet of Things Realize its Potential. Computer.

[21] Chung K., Yoo H., Choe D. (2020). Ambient Context-based Modeling for Health Risk Assessment Using Deep Neural Network. J. Ambient Intell. Humaniz. Comput..

[22] Adhikari S.P., Yang C., Slot K., Strzelecki M., Kim H. (2018). Hybrid No-Propagation Learning for Multilayer Neural Networks. Neurocomputing.

[23] Kim J.C., Chung K. (2019). Prediction Model of User Physical Activity using Data Characteristics-based Long Short-term Memory Recurrent Neural Networks. KSII Trans. Internet Inf. Syst..

[24] Baek J.W., Chung K. (2020). Context Deep Neural Network Model for Predicting Depression Risk using Multiple Regression. IEEE Access.

[25] Fischer T., Krauss C. (2018). Deep Learning with Long Short-term Memory Networks for Financial Market Predictions. Eur. J. Oper. Res..

[26] Kim J.C., Chung K. (2018). Mining Health-Risk Factors using PHR Similarity in a Hybrid P2P Network. Peer Peer Netw. Appl..

[27] Baek J.W., Kim J.C., Chun J.C., Chung K. (2019). Hybrid Clustering based Health Decision-making for improving Dietary Habits. Technol. Health Care.

[28] Kim J.C., Chung K. (2020). Neural-Network based Adaptive Context Prediction Model for Ambient Intelligence. J. Ambient Intell. Humaniz. Comput..

[29] Jung H., Chung K. (2016). Life Style Improvement Mobile Service for High Risk Chronic Disease based on PHR Platform. Cluster Comput..

[30] Kim J.C., Chung K. (2017). Emerging Risk Forecast System using Associative Index Mining Analysis. Cluster Comput..

[31] Yoo H., Chung K. (2018). Heart Rate Variability based Stress Index Service Model using Bio-Sensor. Cluster Comput..

[32] Chung K., Na Y., Lee J.H. (2013). Interactive Design Recommendation using Sensor based Smart Wear and Weather WebBot. Wirel. Pers. Commun..

[33] Yoo H. (2019). Health Big Data Processing Method Based on Deep Neural Network for Preventing Cardiovascular Disease. Ph.D. Thesis.

[34] Sathyapriya L., Murail L., Manigandan T. Analysis and Detection R-peak Detection Using Modified Pan-Tompkins Algorithm. Proceedings of the 2014 IEEE International Conference on Advanced Communications, Control and Computing Technologies.

[35] Yao J., Warren S. (2006). Applying the ISO/IEEE 11073 Standards to Wearable Home Health Monitoring Systems. J. Clin. Monit. Comput..

[36] Yoo H., Chung K. (2018). Mining-based Lifecare Recommendation using Peer-to-Peer Dataset and Adaptive Decision Feedback. Peer Peer Netw. Appl..

[37] Canziani A., Paszke A., Culurciello E. (2017). An Analysis of Deep Neural Network Models for Practical Applications. arXiv.

[38] Sugomori Y. (2016). Java Deep Learning Essentials.

[39] Kang J.S., Baek J.W., Chung K. (2020). PrefixSpan based Pattern Mining using Time Sliding Weight for Streaming Data. IEEE Access.

[40] Shin D.H., Park R.C., Chung K. (2020). Decision Boundary-Based Anomaly Detection Model using Improved AnoGAN from ECG Data. IEEE Access.

[41] Kim J.C., Chung K. (2020). Multi-modal Stacked Denoising Autoencoder for Handling Missing Data in Health Big Data. IEEE Access.

